# Dentoskeletal characteristics of non-syndromic pierre robin sequence and isolated incomplete cleft palate children: a retrospective case control study

**DOI:** 10.3389/fped.2025.1519266

**Published:** 2025-02-24

**Authors:** Xiang Zhang, Shuang Yang, Xudong Yang, Zhibo Zhou

**Affiliations:** ^1^ Department of Anesthesiology, Peking University School and Hospital of Stomatology, Beijing, China; ^2^ Department of Oral and Maxillofacial Surgery, Peking University School and Hospital of Stomatology, Beijing, China

**Keywords:** non-syndromic pierre robin sequence, cleft palate, micrognathia, dentoskeletal characteristics, pediatric

## Abstract

**Background:**

Pierre Robin sequence (PRS) is characterized by micrognathia, glossoptosis, and upper airway obstruction. This study aimed to compare the dentoskeletal characteristics of children diagnosed with non-syndromic PRS and those with cleft palate.

**Methods:**

This study was conducted on the non-syndromic PRS patients in the database of our hospital. The control group of non-syndromic isolated incomplete cleft palate patients was matched from the same database by age, gender and weight in a 1:3 ratio. The dentoskeletal characteristics were compared between the PRS and control groups.

**Results:**

The study included 14 patients in the PRS group and 42 patients in the control group. A point-Nasion-B point (ANB) angle was significantly greater in the PRS group compared with the control group. The PRS group exhibited a significantly lower ratio of the linear distance between Articulare and Gonion (ArGo) to the distance between Gonion and Pogonion (GoPo) compared to the control group. Additionally, the anteroposterior length and area of the lower pharyngeal airway space (LPAS) were markedly reduced in the PRS group.

**Conclusion:**

The ANB angle, the ArGo/GoPO ratio and the anteroposterior length and area of LPAS could serve as valuable indicators to identify micrognathia in patients with non-syndromic PRS.

## Introduction

Pierre Robin sequence (PRS) is characterized by micrognathia, glossoptosis, and often airway compromise, which is frequently accompanied by a U-shaped cleft palate of varying severity, occurring in approximately 1 in 10,000 births (1). The sequence is termed non-syndromic PRS when it occurs without craniofacial syndrome (2). Micrognathia reduces tongue muscle support, leading to its descent into the subpharyngeal space, where it forms a spherical valve. While this valve permits exhalation, it obstructs inhalation, resulting in dyspnea as a significant manifestation of PRS (3). Micrognathia is typically defined as a visibly smaller mandibular body with mental retrusion (4). Identifying micrognathia from various perspectives is crucial for comprehensive patient care. Dental professionals can provide timely orthodontic intervention (1). Anesthesiologists need to be aware of micrognathia for effective airway management during intubation. Additionally, its impact on respiratory function necessitates monitoring and treatment of diseases such as obstructive sleep apnea (3).

However, not all visually diagnosed cases exhibit clinical symptoms. Researchers are divided on the extent to which the mandible undergoes proportional reduction before the growth spurt (5), with some suggesting a proportional reduction while others argue against it, citing ratios like ramus length to mandibular length (6). Certain aspects of this topic remain inconclusive. However, the quantitative characteristics of the maxillofacial region in PRS patients are not clearly understood.

Three-dimensional (3D) reconstruction using Mimics software involves converting two-dimensional medical images, such as computed tomography (CT) or magnetic resonance imaging (MRI), into detailed 3D models of anatomical structures. The Mimics software utilizes segmentation algorithms to delineate specific tissues or structures, allowing for accurate reconstruction and visualization. The advantages of its application in studies involving micrognathia include precise evaluation of craniofacial morphology, and enhanced surgical planning through virtual simulations. The Mimics software employs advanced segmentation algorithms to delineate specific tissues or structures, enabling accurate reconstruction and visualization. This capability allows clinicians to analyze complex anatomical relationships and assess treatment outcomes more effectively, ultimately leading to more personalized and effective patient care in the management of micrognathia (7).

Distinguished from other studies (8–10), quantitative distinctions in mandibular characteristics were identified by comparing certain alternative airway and mandibular parameters through 3D reconstruction using Mimics software. Given the absence of normal pediatric head CT data, the present study selected patients with non-syndromic isolated incomplete cleft palate (11) and normal mandibles as the control group. The objective of the present study was to compare the dentoskeletal characteristics of children diagnosed with non-syndromic PRS and those with cleft palate.

## Materias and methods

### Study design and patients

This study retrospectively retrieved the database of the Peking University School and Hospital of Stomatology between January 2017 and December 2019 using the following keywords: “Robin syndrome,” “PR syndrome,” “Pierre Robin Sequence,” “PRS,” and “U-shaped cleft palate + micrognathia.” Patients diagnosed with PRS upon admission or presenting with a combination of U-shaped cleft palate, micrognathia, and symptoms of glossoptosis were included (10, 12, 13). The exclusion criteria were as follows: the presence of syndromic symptoms, congenital heart disease or a history of surgery for congenital heart disease, a combination of PRS with accessory tragus or polydactyly, a history of undergoing mandibular distraction osteogenesis, or incomplete data. Diagnosing non-syndromic isolated incomplete cleft palate involves a thorough clinical evaluation, where a healthcare professional examined the palate for gaps that do not extend into the lip or nose and in patients without other abnormalities (11). The CT scan was conducted before cleft palate repair. Control patients with other comorbidities or incomplete data were excluded. This study adhered to the principles of the Declaration of Helsinki in terms of medical protocols and ethics and was approved by the Institutional Ethical Committee of Peking University School and Hospital of Stomatology (PKUSSIRB-201950149). This article is a retrospective study. Therefore the Institutional Ethical Committee of Peking University School and Hospital of Stomatology waived the requirement to obtain distinct written informed consent from the patients.

Patients with non-syndromic isolated incomplete cleft palate were selected as the control group at a 1:3 ratio in accordance with similar studies (14, 15). The following assumptions were made to calculate the sample size: the ANB of the PRS group was expected to be 30% higher than that of the control group. According to our preliminary pilot study (unpublished) with 10 non-syndromic PRS cases, the ANB of non-syndromic PRS was 8.72 ± 2.29. With *α* and *β* errors set at 5% each, a power analysis conducted using PASS 21 (NCSS, Kaysville, UT) indicated that 14 cases would be needed for the PRS group and 42 for the control group. Control group cases were matched based on age, gender, and weight.

### Data collection and processing of CT images

Spiral CT images of the patients (with a layer thickness of 1.25 mm; brightSpeed16, GE Healthcare, Buckinghamshire, UK) were obtained using their medical record numbers. The raw data were stored in the DICOM (Digital Imaging and Communications in Medicine) format. Image processing involved importing the DICOM format data into Mimics 21.0 software. The horizontal plane was aligned with the orbital ear plane (Frankfort Horizontal Plane, PO). For airway analysis, the final measurement was limited to the plane from the upper edge of the first cervical vertebra to the lower edge of the fourth cervical vertebra. The Split Mask tool was utilized to isolate the mandible, followed by using the Edit Mask tool to remove any excess portions. Reconstruction of the 3D image of the airway and mandible was carried out using the Calculate-in-3D tool. The CT data underwent re-analysis by the same experienced examiner with over 10 years of expertise in oral radiation medicine, involving repeated reconstructions at a 2-week interval. Calibration points were defined to standardize measurements of various parameters, including linear distances, planes, and angles related to dentoskeletal characteristics. The definitions of calibration points are presented in Table 1. Demographic characteristics of age, gender, and weight were collected from the database of the hospital. The data collection process was conducted by experienced personnel with expertise in oral radiation medicine, ensuring accuracy and reliability.

**Table 1:** Definition of the calibration point.

Parameter	Definition
Landmark	
N, Nasion	Most anterior point on fronto-nasal suture
S, Sella	Geometric center of pituitary fossa
P, Porion	Top point of external auditory canal
Or, Orbitale	The lowest point of infraorbital margin
A. Subspinale	The most concave point of the bone between the anterior nasal spine and the upper alveolar edge point
B, Supramental	The most concave point of the bone between the lower alveolar edge point and the premental point
Po, Pogonion	The most prominent point of chin
Gn, Gnathion	The midpoint of the bone junction between the premental point and the submental point
Go, Gonion	Posterior inferior point of mandibular angle
Ar, Articulare	The intersection of the lower edge of the skull base and the posterior edge of the mandibular condyle neck
Me, Menton	The lowest point of chin
Distance	
Go-Po	The linear distance between Go and Po
Ar-Go	The linear distance between Ar and Go
Anteroposterior length of LPAS	The distance from the most posterior border of the tongue base to the most posterior point on wall of the pharynx at that level
Transversal length of LPAS	The distance from the rightmost edge to the leftmost edge of the pharyngeal walls at the level
Plane	
Frankfort Horizontal Plane, PO	A plane composed of point P and point O
Mandibular Plane, MP	A plane composed of point Me and point Go
Ramal Plane, RP	Tangent line between Ar and Posterior margin of mandibular angle
Lower Pharyngeal Airway Space, LPAS	The location with the horizontal shortest distance between the posterior border of the tongue base and the posterior pharyngeal wall on sagittal section
Area	
Area of LPAS	The airway area at LPAS level
Angle	
Full soft tissue convexity, FSTC	The Angle formed by point Ns, point Prn and point Pos
FMA	The angle between the Frankfort Horizontal Plane and the mandibular plane
SNB angle	The Angle formed by point S, point N and point B
ANB angle	The Angle formed by point A, point N and point B
NSGn angle	The Angle formed by point N, point S and point Gn
Gonial angle	The Angle formed by MP and RP

### Statistical analysis

The statistical analysis was conducted through SPSS 26.0 software (IBM, Armonk, NY, USA). For quantitative data, descriptive statistics, including median and range were reported, while categorical data were described as frequency and percentage. Inter-group comparisons of weights, age, and average cephalometric parameters were performed using independent samples *t*-tests. The reliability of measurements was ensured by conducting repeated assessments by the same researcher at 2-week intervals. The intraclass correlation coefficient (ICC) were used analyze the consistency of the two measurements. An ICC value exceeding 0.90 indicated satisfactory agreement between the initial and subsequent measurements. A two-sided *P* < 0.05 was considered as statistically significance.

## Results

**Figure 1:**
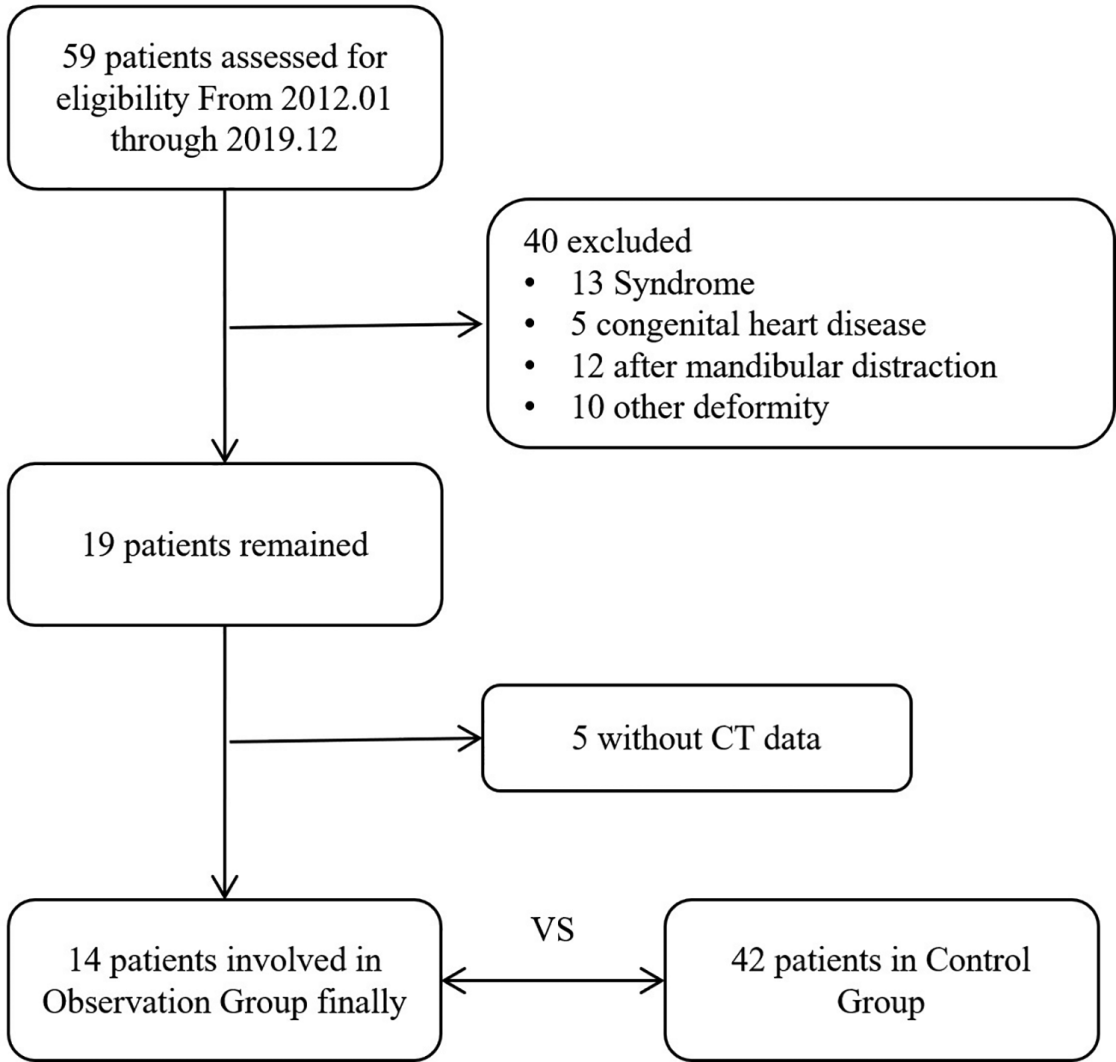
The flowchart.

**Figure 2:**
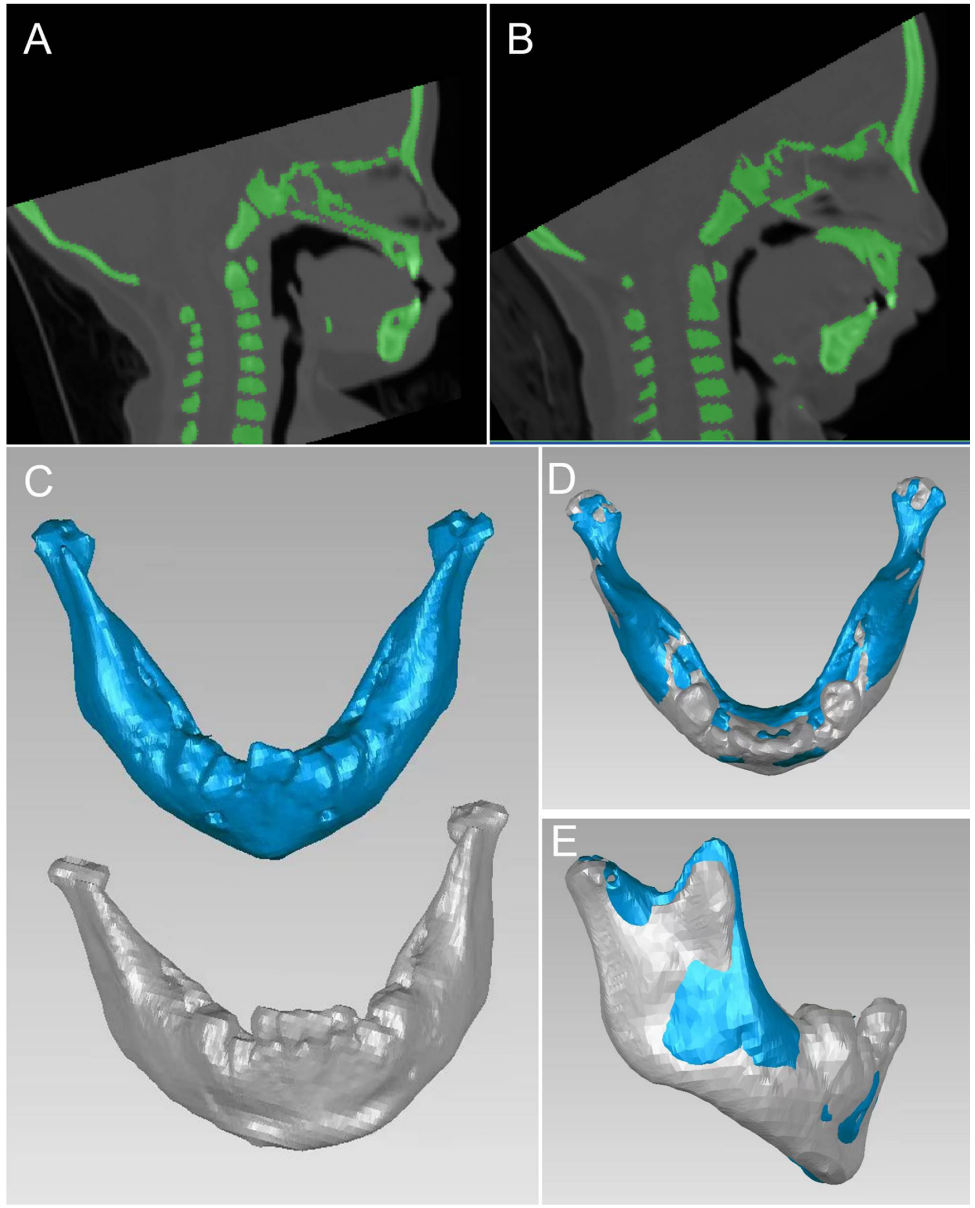
2D contrast of control group **(A)** and PRS group **(B)** 3D contrast of mandible **(C–E)**.

**Figure 3:**
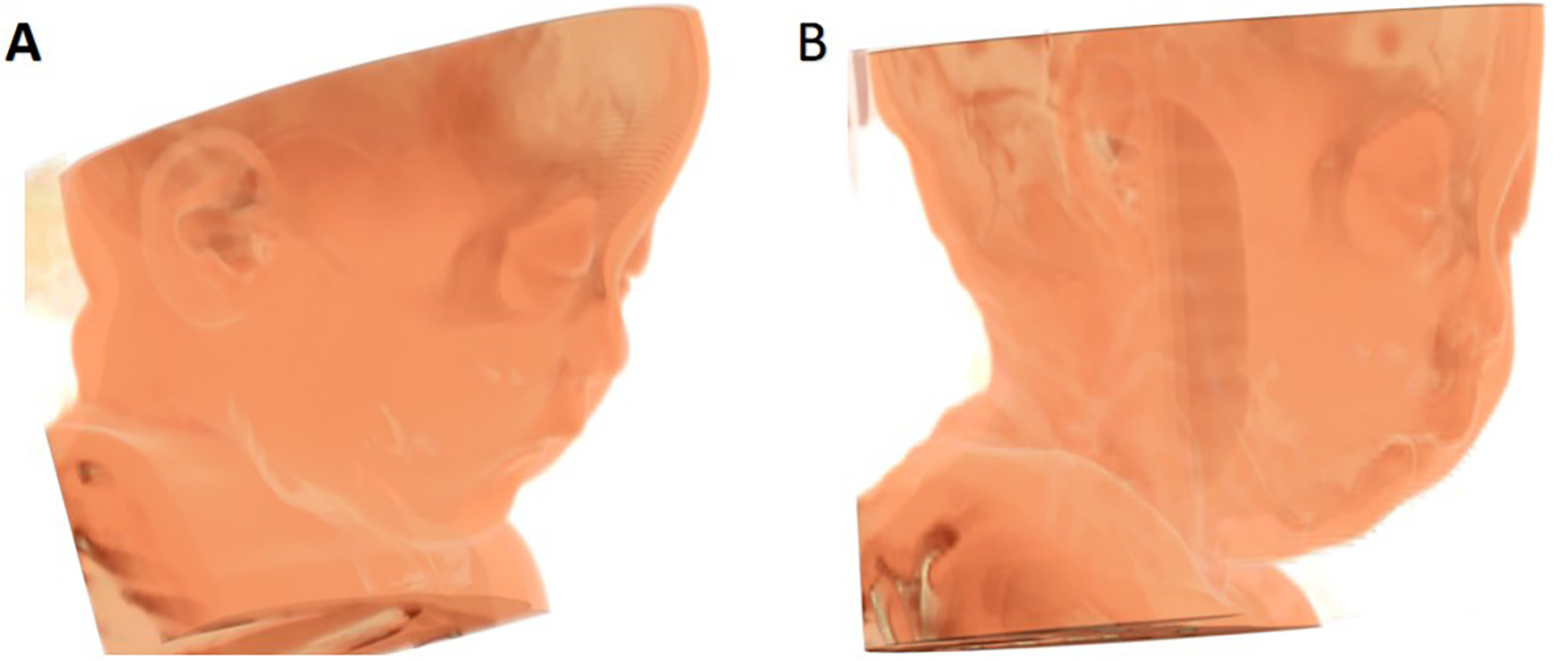
3D contrast of facial soft tissue reconstruction between control group **(A)** and PRS group **(B).**

Initially, 59 PRS related cases were identified. Subsequently, cases were excluded based on predefined criteria, such as the presence of syndromic presentations (*n* = 13), patients with congenital heart disease or a history of undergoing surgery for congenital heart disease (*n* = 5), a combination of PRS with accessory tragus or polydactyly (*n* = 10), patients with a history of undergoing mandibular distraction osteogenesis (*n* = 12), and patient with incomplete electronic CT data (*n* = 5). As a result, a final sample of 14 PRS cases was finally included. 42 patients with isolated incomplete cleft palate were selected as the control group. The patient selection process is illustrated in Figure 1. The 3D reconstruction of dentoskeletal structures and facial soft tissue using Mimics software for both the PRS group and the control group was presented in Figures 2, 3.

The paired sample *t*-test indicated no significant difference between the first and second measurements in the repeated data (all *P* > 0.05). Additionally, an ICC value exceeding 0.90 demonstrated strong consistency among the experimental results (Supplementary Table S1).

No significant difference in age, gender and body weight was found between the PRS and control groups (Table 2). A point-Nasion-B point (ANB) angle was significantly greater in the PRS group compared with the control group (9.59 ± 3.51 vs. 5.45 ± 1.43, *P* = 0.002). The ratio of the linear distance between Articulare and Gonion (ArGo) to the linear distance between Gonion and Pogonion (GoPo) was significantly lower in the PRS group compared to the control group (0.48 ± 0.07 vs. 0.49 ± 0.03, *P* = 0.008). Additionally, the anteroposterior length (6.26 ± 2.28 vs. 8.96 ± 2.73 mm, *P* = 0.001) and area（75.74 ± 25.18 vs. 90.98 ± 27.68mm^2^, *P* = 0.036）of the lower pharyngeal airway space (LPAS) were markedly reduced in the PRS group. The mandible volume, upper airway volume, full soft tissue convexity, Sella-Nasion-B point angle, Nasion-Sella-Gnathion angle, Frankfort-mandibular angle, gonial angle, ArGo, and GoPo exhibited no significant differences between the two groups (all *P* > 0.05) (Table 3).

**Table 2:** Comparison of demographic characteristics between the two groups.

Demographic characteristics	PRS group (*n* = 14)	Control group (*n* = 42)	*P*
Gender, *n* (%)			0.999
Male	12 (21.43)	36 (64.28)	
Female	2 (3.57)	6 (10.71)	
Weight (kg)	9.93 ± 1.52	9.71 ± 1.54	0.636
Age (months)	10.50 ± 1.16	10.57 ± 1.05	0.812

**Table 3:** Comparison of clinical characteristics between the PRS and control groups.

Clinical characteristics	PRS group	Control group	*P*
Mandible volume (mm^3^)	19,206.07 ± 1,375.00	21,511.96 ± 4,258.49	0.094
Upper airway volume (mm^3^)	2,121.13 ± 472.85	2,579.73 ± 735.78	0.148
FSTC	138.36 ± 5.09	142.43 ± 4.08	0.181
SNB angle	61.96 ± 2.02	72.19 ± 3.86	0.089
ANB angle*	9.59 ± 3.51	5.45 ± 1.43	0.002
NSGn angle	75.12 ± 4.07	74.59 ± 3.21	0.286
FMA	38.84 ± 5.56	38.55 ± 6.67	0.084
Gonial angle	131.46 ± 3.16	133.67 ± 5.50	0.110
ArGo	24.96 ± 3.30	26.08 ± 1.93	0.236
GoPo	51.69 ± 3.45	52.65 ± 3.19	0.170
ArGo/GoPo*	0.48 ± 0.07	0.49 ± 0.03	0.008
Anteroposterior length of LPAS (mm)*	6.26 ± 2.28	8.96 ± 2.73	0.001
Transversal length of LPAS (mm)	11.04 ± 4.66	10.18 ± 3.21	0.424
Area of LPAS (mm^2^)	75.74 ± 25.18	90.98 ± 27.68	0.036

FSTC, full soft tissue convexity; SNB, Sella-Nasion-B point; ANB, a point-Nasion-B point; NSGn, Nasion-Sella-Gnathion; FMA, Frankfort-Mandibular angle; ArGo, ArGo point; GoPo, GoPo point; LPAS, Lower Pharyngeal Airway Space.

*P < 0.05.

## Discussion

This study revealed that the ANB angle, the ArGo/GoPO ratio and the anteroposterior length and area of LPAS could be valuable indicators for identifying micrognathia in patients with non-syndromic PRS. These parameters may represent potential markers for early diagnosis and monitoring of patient progress, warranting validation in further studies and clinical practice.

The ArGo/GoPo ratio of the PRS group was smaller than that of the control group, suggesting a disproportionate reduction in ArGo and GoPo. The mandibular ascending branch and the mandibular length exhibited disproportionate growth in the PRS group, with the mandibular ascending branch displaying slower development (6). The mandibular retraction and overall underdevelopment contributed to a narrow retro-lingual airspace. Mao and Ye (16) assessed the 3D changes in airway size and shape in newborns with isolated PRS who underwent mandibular distraction osteogenesis. They concluded that mandibular distraction osteogenesis in isolated PRS cases could enhance the size and configuration of the upper airway, confirming its effectiveness as a surgical intervention for alleviating airway obstruction in newborns with isolated PRS. Prior research (17) assessed the phonatory and morphological outcomes of 72 cognitively unimpaired adolescents with PRS. Morphological or phonatory impairments persisted in adolescents with PRS, although they did not appear to directly affect their quality of life. Adolescents, particularly those with non-isolated PRS, exhibited fragility in self-confidence and social relationships. European scholars assessed survival, hospitalization, and surgical procedures for children born with PRS across Europe (18). They found that infants with PRS experienced elevated mortality and morbidity rates, resulting in prolonged hospitalizations during their first year of life, with nearly all undergoing surgery before the age of five. However, survival rates improved beyond infancy, leading to decreased hospital admissions after reaching five years of age. Following cleft palate surgery, alterations in the patient's oropharynx volume within the airway are inevitable (19–21). Post-palatoplasty, the oropharyngeal airway mainly demonstrates transverse narrowing due to soft palate closure (22–24). Techniques, such as Fulow palatoplasty may further reduce transverse pharyngeal dimensions to achieve palatal elongation (25–27). Indirect evidence, such as postoperative stertor, suggests a reduction in patients' postoperative airway volume (28). Prior research highlighted that the original upper airway volume of children with PRS was lower than that of patients with similar clefts lacking micrognathia (29), alerting anesthesiologists to the elevated incidence of postoperative airway disorders in PRS patients. In a guideline for surgical management of neonates with PRS or severe micrognathia (30), CT was utilized to assess the structure and positioning of the tongue, as well as to delineate the relative placement of the inferior alveolar nerve upon entry into the mandible. Additionally, a CT scan aided in identifying the position of tooth buds and quantifying the minimum airway space (31). Han et al. (32) documented the utilization of allogenic acellular bone matrix and mandibular distraction osteogenesis in PRS cases and investigated the impact of distraction on the osteogenesis of acellular bone. They found that combining bilateral mandibular distraction osteogenesis with the placement of allogenic acellular bone in neonates represented safe and precise procedures, serving as the primary treatment modalities for severe PRS cases (32).

This study focused on exploring the digital diagnosis of micrognathia in non-syndromic PRS patients by integrating existing parameters established by other researchers. Our goal is to enhance the performance of digital models and provide valuable insights to the broader community. Digital models with high specificity indicators, combined with patient history, enable stomatologists and anesthesiologists to perform a more quantitative assessment of the airway before surgery, as opposed to relying solely on visual examination. This method is especially crucial developing countries.

The limitations of the present study should be pointed out. Firstly, the single-center, retrospective design of the study might introduce potential biases and incomplete data, such as missing information on head circumference, chest circumference, and head-to-body ratio. Secondly, the small sample size, particularly the reduced sample size for PRS due to missing electronic CT data, might limit the generalizability of the findings and increase the risk of type II errors. Finally, the study primarily concentrated on quantitative dentoskeletal characteristics, while qualitative aspects, such as functional outcomes or patient-reported measures were not assessed. Further large-scale prospective studies are warranted to overcome these limitations and provide more robust evidence.

In conclusion, this study concentrated on the dentoskeletal characteristics of non-syndromic PRS children compared with those with cleft palates, utilizing advanced imaging techniques and sophisticated software for analysis. The findings revealed significant differences in certain cephalometric parameters, particularly the ANB angle, the ArGo/GoPo ratio, and the anteroposterior length and area of LPAS between the PRS and control groups, highlighting the potential utility of these metrics as indicators for diagnosing micrognathia in non-syndromic PRS patients. Future research is required to validate these parameters.

## Data Availability

The original contributions presented in the study are included in the article/Supplementary Material, further inquiries can be directed to the corresponding author.

## References

[1] Gangopadhyay N Mendonca DA Woo AS. Pierre robin sequence. Semin Plast Surg. (2012) 26(2):76–82. 10.1055/s-0032-1320065 23633934 PMC3424697

[2] Xu JX Kilpatrick N Baker NL Penington A Farlie PG Tan TY. Clinical and molecular characterisation of children with pierre robin sequence and additional anomalies. Mol Syndromol. (2016) 7(6):322–8. 10.1159/000449115 27920635 PMC5131331

[3] Morokuma S Anami A Tsukimori K Fukushima K Wake N. Abnormal fetal movements, micrognathia and pulmonary hypoplasia: a case report. Abnormal fetal movements. BMC Pregnancy Childbirth. (2010) 10:46. 10.1186/1471-2393-10-46 20716376 PMC2931455

[4] Weaver KN Sullivan BR Balow SA Hopkin S Chini BA Pan BS Robin sequence without cleft palate: genetic diagnoses and management implications. Am J Med Genet A. (2022) 188(1):160–77. 10.1002/ajmg.a.62515 34569146

[5] Morrison KA Collares MV Flores RL. Robin sequence: neonatal mandibular distraction. Clin Plast Surg. (2021) 48(3):363–73. 10.1016/j.cps.2021.03.005 34051891

[6] Logjes RJH Breugem CC Van Haaften G Paes EC Sperber GH van den Boogaard MH The ontogeny of Robin sequence. Am J Med Genet A. (2018) 176(6):1349–68. 10.1002/ajmg.a.38718 29696787

[7] Yao J Dong B Sun J Liu JT Liu F Li XW Accuracy and reliability of computer-aided anatomical measurements for vertebral body and disc based on computed tomography scans. Orthop Surg. (2020) 12(4):1182–9. 10.1111/os.12729 32618427 PMC7454159

[8] Suri S Ross RB Tompson BD. Mandibular morphology and growth with and without hypodontia in subjects with Pierre Robin sequence. Am J Orthod Dentofacial Orthop. (2006) 130(1):37–46. 10.1016/j.ajodo.2005.09.026 16849070

[9] Brockmeyer P Wiechens B Sevinc T Schliephake H Hahn W. Informational content of two-dimensional panoramic radiographs and lateral cephalometric radiographs with respect to the bone volume of intraoral donor regions considering Cbct imaging. BMC Oral Health. (2022) 22(1):318. 10.1186/s12903-022-02344-6 35907826 PMC9339174

[10] Kato RM Moura PP Zechi-Ceide RM Tonello C Peixoto AP Garib D. Comparison between treacher collins syndrome and Pierre Robin sequence: a cephalometric study. Cleft Palate Craniofac J. (2021) 58(1):78–83. 10.1177/1055665620937499 32613853

[11] Mossey PA Little J Munger RG Dixon MJ Shaw WC. Cleft lip and palate. Lancet. (2009) 374(9703):1773–85. 10.1016/S0140-6736(09)60695-4 19747722

[12] Hanson JW Smith DW. U-shaped palatal defect in the Robin Anomalad: developmental and clinical relevance. J Pediatr. (1975) 87(1):30–3. 10.1016/s0022-3476(75)80063-1 1151545

[13] Baujat G Faure C Zaouche A Viarme F Couly G Abadie V. Oroesophageal motor disorders in Pierre Robin syndrome. J Pediatr Gastroenterol Nutr. (2001) 32(3):297–302. 10.1097/00005176-200103000-00012 11345179

[14] Ben Abdallah I Craiem D Casciaro M Deza D Ronot M Corcos O Case-Control study of 3d morphology in isolated mesenteric artery dissection. Cardiovasc Eng Technol. (2023) 14(2):230–8. 10.1007/s13239-022-00649-9 36471224

[15] Wu WF Yi JS Xie X Liu CB. Risk factor for interstitial pregnancy following ipsilateral salpingectomy? A retrospective matched case control study. BMC Pregnancy Childbirth. (2023) 23(1):826. 10.1186/s12884-023-06132-0 38037027 PMC10687775

[16] Mao Z Ye L. Effects of mandibular distraction osteogenesis on three-dimensional upper airway anatomy in newborns affected by isolated pierre robin sequence. J Craniofac Surg. (2021) 32(4):1459–63. 10.1097/SCS.0000000000007339 34403227

[17] Thouvenin B Soupre V Caillaud MA Henry-Mestelan C Chalouhi C Houssamo B Quality of life and phonatory and morphological outcomes in cognitively unimpaired adolescents with pierre robin sequence: a cross-sectional study of 72 patients. Orphanet J Rare Dis. (2021) 16(1):442. 10.1186/s13023-021-02072-0 34670591 PMC8527704

[18] Santoro M Garne E Coi A Tan J Loane M Ballardini E Survival, hospitalisation and surgery in children born with pierre robin sequence: a European population-based cohort study. Arch Dis Child. (2023) 108(7):550–5. 10.1136/archdischild-2022-324716 37160334 PMC10314079

[19] Perez FA Hottinger DG Evans KN Giles M Otto RK Hunyady A Longer upper airway lengths in Robin sequence: a case-control study using computed tomography. Paediatr Anaesth. (2020) 30(6):683–90. 10.1111/pan.13869 32277728

[20] Kosyk MS Carlson AR Zapatero ZD Kalmar CL Swanson JW Bartlett SP Cleft palate repair in robin sequence following mandibular distraction osteogenesis compared to tongue-lip adhesion. Cleft Palate Craniofac J. (2023) 60(2):151–8. 10.1177/10556656211055019 34730034

[21] Santoro M Coi A Barišić I Pierini A Addor MC Baldacci S Epidemiology of pierre-robin sequence in Europe: a population-based eurocat study. Paediatr Perinat Epidemiol. (2021) 35(5):530–9. 10.1111/ppe.12776 34132407

[22] Hardwicke JT Richards H Cafferky L Underwood I Horst BT Slator R. Outcomes of cleft palate repair in patients with pierre robin sequence: a matched case-control study. Plast Reconstr Surg. (2016) 137(3):927–35. 10.1097/01.prs.0000475829.32402.a8 26910675

[23] Giudice A Barone S Belhous K Morice A Soupre V Bennardo F Pierre robin sequence: a comprehensive narrative review of the literature over time. J Stomatol Oral Maxillofac Surg. (2018) 119(5):419–28. 10.1016/j.jormas.2018.05.002 29777780

[24] Côté A Fanous A Almajed A Lacroix Y. Pierre robin sequence: review of diagnostic and treatment challenges. Int J Pediatr Otorhinolaryngol. (2015) 79(4):451–64. 10.1016/j.ijporl.2015.01.035 25704848

[25] Kosyk MS Carlson AR Zapatero ZD Kalmar CL Swanson JW Bartlett SP Cleft palate repair in Robin sequence following mandibular distraction osteogenesis compared to tongue-lip adhesion. Cleft Palate Craniofac J. (2023) 60(2):151–8. 10.1177/10556656211055019 34730034

[26] Zaballa K Singh J Waters K. The management of upper airway obstruction in pierre robin sequence. Paediatr Respir Rev. (2023) 45:11–5. 10.1016/j.prrv.2022.07.001 35987882

[27] Morris JK Garne E Loane M Barisic I Densem J Latos-Bieleńska A Eurolinkcat protocol for a European population-based data linkage study investigating the survival, morbidity and education of children with congenital anomalies. BMJ Open. (2021) 11(6):e047859. 10.1136/bmjopen-2020-047859 34183346 PMC8240574

[28] Pimenta LA de Rezende Barbosa GL Pretti H Emodi O van Aalst J Rossouw PE Three-dimensional evaluation of nasopharyngeal airways of unilateral cleft lip and palate patients. Laryngoscope. (2015) 125(3):736–9. 10.1002/lary.24895 25180659

[29] Mao Z Ye L. Effects of mandibular distraction osteogenesis on three-dimensional upper airway anatomy in newborns affected by isolated Pierre Robin sequence. J Craniofac Surg. (2021) 32(4):1459–63. 10.1097/scs.0000000000007339 34403227

[30] Cladis F Kumar A Grunwaldt L Otteson T Ford M Losee JE. Pierre Robin sequence: a perioperative review. Anesth Analg. (2014) 119(2):400–12. 10.1213/ane.0000000000000301 25046788

[31] Soto E Ananthasekar S Kurapati S Robin NH Smola C Maddox MH Mandibular distraction osteogenesis as a primary intervention in infants with pierre robin sequence. Ann Plast Surg. (2021) 86(6S Suppl 5):S545–s9. 10.1097/SAP.0000000000002702 33833161 PMC8601586

[32] Han T Ji Y Cui J Kong L Shi L Chen J Treated pierre robin sequence using placed allogenic acellular bone matrix and mandibular distraction osteogenesis in the neonate. Front Pediatr. (2022) 10:890156. 10.3389/fped.2022.890156 35676894 PMC9168749

