# Supplementary material for: Dentoskeletal characteristics of non-syndromic pierre robin sequence and isolated incomplete cleft palate children: a retrospective case control study
Source: Front Pediatr. 2025 Feb 24;13:1519266. doi: 10.3389/fped.2025.1519266 (PMC11891179; doi:10.3389/fped.2025.1519266)
Supplement: Supplementary file 1 [file Table1.docx]

**Table S1. The credibility analysis**

| **Parameters** | **PRS group** | | **Control group** | |
| --- | --- | --- | --- | --- |
|  | **P** | **ICC** | **P** | **ICC** |
| Mandible volume | 0.253 | 0.966 | 0.145 | 0.995 |
| Upper airway volume | 0.161 | 0.983 | 0.22 | 0.986 |
| FSTC | 0.382 | 0.991 | 0.102 | 0.957 |
| SNB angle | 0.494 | 0.923 | 0.247 | 0.971 |
| ANB angle | 0.092 | 0.996 | 0.103 | 0.983 |
| NSGn angle | 0.143 | 0.943 | 0.137 | 0.996 |
| FMA | 0.275 | 0.937 | 0.111 | 0.964 |
| Gonial angle | 0.06 | 0.958 | 0.392 | 0.982 |
| ArGo | 0.192 | 0.941 | 0.078 | 0.97 |
| GoPo | 0.106 | 0.979 | 0.148 | 0.905 |
| ArGo/GoPo | 0.417 | 0.974 | 0.087 | 0.974 |

Note: FSTC, full soft tissue convexity; SNB, Sella-Nasion-B point; ANB, A point-Nasion-B point; NSGn, Nasion-Sella-Gnathion; FMA, Frankfort-Mandibular angle; ArGo, ArGo point; GoPo, GoPo point; ICC, intraclass correlation coefficient.
